# Predictors of Treatment Outcomes among Multidrug Resistant Tuberculosis Patients in Tanzania

**DOI:** 10.1155/2019/3569018

**Published:** 2019-02-12

**Authors:** Tamary Henry Leveri, Isack Lekule, Edson Mollel, Furaha Lyamuya, Kajiru Kilonzo

**Affiliations:** ^1^Kilimanjaro Christian Medical University College, P.O. Box 2240, Moshi, Tanzania; ^2^Kibong'oto Infectious Diseases Hospital, P.O. Box 12, Sanya Juu, Tanzania; ^3^Western Zone Blood Transfusion Center, P.O. Box 1782, Tabora, Tanzania

## Abstract

**Background:**

According to World Health Organization (WHO) the final multidrug resistant tuberculosis (MDRTB) treatment outcome is the most important direct measurement of the effectiveness of the MDRTB control program. Literature review has shown marked diversity in predictors of treatment outcomes worldwide even among the same continents. Therefore, findings could also be different in Tanzanian context, where the success rate is still lower than the WHO recommendation. This study sought to determine the predictors of treatment outcomes among MDRTB patients in Tanzania in order to improve the success rate.

**Methodology:**

This was a retrospective cohort study, which was conducted at Kibong'oto Infectious Diseases Hospital (KIDH) in Tanzania. Patients' demographic and clinical parameters were collected from the MDRTB registry and clinical files. Then, a detailed analysis was done to determine the predictors of successful and unsuccessful MDRTB treatment outcomes.

**Results:**

Three hundred and thirty-two patients were diagnosed and put on MDRTB treatment during the year 2009 to 2014. Among them, males were 221 (67%), and 317 (95.48%) were above 18 years of age, mean age being 36.9 years. One hundred and sixty-one patients (48.5%) were living in Dar es Salaam. The number of MDRTB patients has increased from 16 in 2009 to 132 in 2014. Majority of patients (75.7%) had successful treatment outcomes. The following predictors were significantly associated with MDRTB cure: presence of cavities in chest X-rays (aOR 1.89, p value 0.002), low BMI (aOR 0.59, p value 0.044), and resistance to streptomycin (aOR 4.67, p value 0.007) and ethambutol (aOR 0.34, p value 0.041). Smoking and presence of cavities in chest X-rays were associated with MDRTB mortality, aOR 2.31, p value 0.043 and aOR 0.55, p value 0.019, respectively.

**Conclusion:**

The study indicated that overall number of MDRTB patients and the proportion of successful treatment outcomes have been increasing over the years. The study recommends improving nutritional status of MDRTB patients, widespread antismoking campaign, and close follow-up of patients with ethambutol resistance.

## 1. Introduction

Drug resistant tuberculosis (DRTB) is a form of tuberculosis (TB), which is resistant to any of the first line antituberculosis drugs. There are different types of DRTB such as monoresistant tuberculosis and multidrug resistant tuberculosis (MDRTB). The former is a form of tuberculosis, which is resistant to any single drug of the first line antituberculosis medications while the latter is resistant to key first line antituberculosis drugs, rifampicin, and isoniazid. Polyresistant tuberculosis is resistant to more than one first line drugs, other than both rifampicin and isoniazid. Extensively Resistant Tuberculosis (XDRTB) includes MDRTB and additional resistance to any of the fluoroquinolones and any of the injectable second line medications.

Among all TB cases worldwide, 3.7% of new cases and 20% of previously treated cases have DRTB [1]. Despite an increased DRTB awareness and roll-out of molecular tests such as Xpert MTB for diagnosis of DRTB, which has enabled early treatment initiation, DRTB and especially MDRTB are still a disease of public health concern. This affects the global efforts to control TB [2].

Globally, there were an estimated 480,000 new cases of MDRTB and an additional 100,000 new cases of rifampicin resistant tuberculosis (RRTB) in the year 2015 [1]. Worldwide the MDRTB/RRTB treatment success rate was only 52%, with 17% mortality [1].

In Tanzania the MDRTB treatment success rate is only 68%, which is lower than that recommended by World Health Organization (WHO) of above 75% [1]. Furthermore, the MDRTB/RRTB incidence has been increasing to 4.9 per 100,000 for year 2015 [1].

According to WHO the final MDRTB treatment outcome is the most important direct measurement of the effectiveness of the MDRTB control program [3]. There are six MDRTB treatment outcomes “cured” (which is restricted to pulmonary TB cases only), “completed”, “died”, “failed”, “lost to follow up”, and “MDRTB cases on MDRTB treatment regimen with no outcome assigned (transferred, still on treatment, or unknown)” [3]. Successful treatment includes being cured and completing treatment, while unsuccessful treatment includes dying, treatment failure, and loss to follow-up.

Cases that have not been evaluated due to transfer or still not completed treatment at the time of final assessment or missing information are all grouped together. Indicators are measured 24 months after the end of the year of assessment to give sufficient time for most patients to complete their treatment and for the final culture results to be issued and retrieved [3].

Treatment of MDRTB is complicated, takes long duration, is associated with several adverse events, and is very expensive [2]. MDRTB treatment is also associated with unsuccessful outcomes compared to non-MDRTB treatment [4]. In the quest to address some of these challenges the WHO has introduced a short treatment regimen for treating MDRTB patients [5].

Tanzania has adopted the new short regimen and has now decentralized MDRTB diagnosis and treatment to the peripheral hospitals which was initially only done at Kibong'oto Infectious Diseases Hospital (KIDH). Factors that have contributed to decentralization include the high cost of treating these patients in inpatients setting and the overwhelming increase in number of MDRTB diagnosis after the introduction and widespread use of Gene Xpert machines for diagnosis of TB in the country. This has led to a substantial increase in early MDRTB diagnosis and early treatment initiation.

Several studies have been done in other countries to determine the predictors of MDRTB treatment outcomes, in the general population and certain specific groups such as those with HIV coinfection, adults, and children [6]. These studies have shown a wide variation in predictors of treatment outcome among countries worldwide.

Some of the predictors that were found to be associated with successful treatment outcomes include Body Mass Index (BMI) >18.5kg/m^2^, use of more than 4 effective drugs, negative baseline sputum smear, and undergoing a surgical resection [7]. Others were the use of fluoroquinolones, or linezolid [8], use of individualized treatment [9], receiving any assistance from TB program, better TB knowledge, and higher level of trust and support from nurses and doctors [10]. Moreover, other studies have focused on the predictors of unsuccessful treatment outcomes, such as extensive drug resistance [8, 11, 12], low BMI [13], hypoalbuminemia [11], comorbidities such as diabetes [11, 14], and cavitary disease [11, 13]. Others were HIV coinfection, positive smear at the start of treatment, and previous history of TB treatment [4, 11, 15], smoking [16, 17], pre-XDRTB, and diabetes mellitus [17],

Other factors include age >44 years [13], resistance to Ofloxacin [8, 15, 18], male sex [16, 19], low body weight at diagnosis < 40 kg [18, 19], and poor drug adherence [19]. Furthermore, smear positivity at month 2 of treatment, use of traditional medicine, and interruption of treatment more than 14 days [14] were also associated with unsuccessful treatment outcome.

In Phillipines, Tupasi and colleagues found that non-HIV immunosuppression, alcohol consumption, lifestyle factors, noncompliance, deficient health education, diabetes, self-rating, and severity of vomiting were the predictors of poor treatment outcome (Loss to follow-up). Other predictors found by other authors include illicit drug use, site of TB [16], ambulatory treatment initiation, having different providers of intensive phase and continuation phase, culture conversion after 4 months [20], and rural residence [18].

However, several studies have shown contradictory findings for some of the factors mentioned above; for example, positive baseline smear and HIV infections were not associated with unsuccessful treatment outcomes [19]. This study aimed to determine the proportions of treatment outcomes and their predictors among MDRTB patients in Tanzania in order to improve the treatment outcomes by combating the predictors or adjusting management protocols accordingly.

## 2. Methodology

This was a retrospective cohort study. It was done from August 2017 to June 2018. It was conducted at KIDH which is located in Siha District in Kilimanjaro region. It is the national specialized public reference hospital assigned for management of MDRTB patients in Tanzania. All patients who were diagnosed with MDRTB since 2009 in Tanzania mainland and Zanzibar were being treated for 18 to 24 months and admitted at KIDH for the whole duration of initial treatment phase (6 to 8 months depending on sputum culture conversion) until recently when decentralization commenced.

Medical records of all MDRTB patients admitted at KIDH from November 2009 (when the MDRTB program started in Tanzania) to December 2014 were studied. The selection of this timeline was based on the availability of the treatment outcomes as the management of MDRTB by then was 18 to 24 months depending on sputum culture conversion. Sputum culture conversion was defined as two consecutive negative culture results. These patients were treated with the standardized regimen, which included (for the intensive phase of minimum 6 months, or 6 months after culture conversion) Amikacin or Kanamycin, Ofloxacin or Levofloxacin, Pyrazinamide, Ethionamide, Cycloserine, and Ethambutol. Then continuation phase of minimum 12 months or 18 months after culture conversion included Ofloxacin or Levofloxacin, Ethionamide, Pyrazinamide, Cycloserine, and Ethambutol. Therefore, the outcomes were available two years after finishing treatment, so that proper treatment outcomes can be assigned to the patient. Excluded patients were those who died before initiation of MDRTB treatment and those who were initially diagnosed with MDRTB but were later found to have* Mycobacterium* other than tuberculosis (MOTT).

### 2.1. Data Collection Method and Tools

Data was collected from the DRTB registry for all patients who were admitted in the KIDH, between November 2009 and December 2014. Data missing in the registry were completed with data from the patients' clinical files. Data collection tool was a data extraction sheet for filling patient's demographic and clinical data.

Study variables were selected in the light of previous studies as shown in the literature review, availability of data, and management protocols in Tanzania context. Available data for the following variables was collected: age, sex, residence, HIV status, history of previously treated TB, smoking status, alcohol use, drug resistance pattern, and the presence of cavities on chest x-rays which was determined by a specialist radiologist. It also included data on baseline BMI, baseline sputum smear and baseline sputum culture.

Treatment outcomes were standardized, as recommended by WHO, as “cured, treatment completed, died, lost to follow-up, and treatment failure” (WHO, 2013). Successful treatment outcomes included declared cured and treatment completion. Unsuccessful treatment outcomes included loss to follow-up, death, and treatment failure.

### 2.2. Data Analysis Plan

Data was then deidentified and entered in an excel sheet. It was then transferred to a statistical software STATA version 14.2 for analysis. Categorical data were summarized as frequencies and percentages. Continuous data was summarized as mean and standard deviation if normally distributed or median and interquartile range if skewed. Statistical significance was considered at 0.05 level. Predictors of successful treatment outcomes and unsuccessful treatment outcomes were then determined, using chi square test. Logistic regression was used to identify predictors which were independently associated with the treatment outcomes.

### 2.3. Ethical Considerations

Ethical clearance was sought from Kilimanjaro Christian Medical University College (KCMUCo) Research Ethical Committee, as well as permission to conduct the study from the KIDH administration. Patients' confidentiality and privacy were strictly observed.

## 3. Results

**Figure 1:**
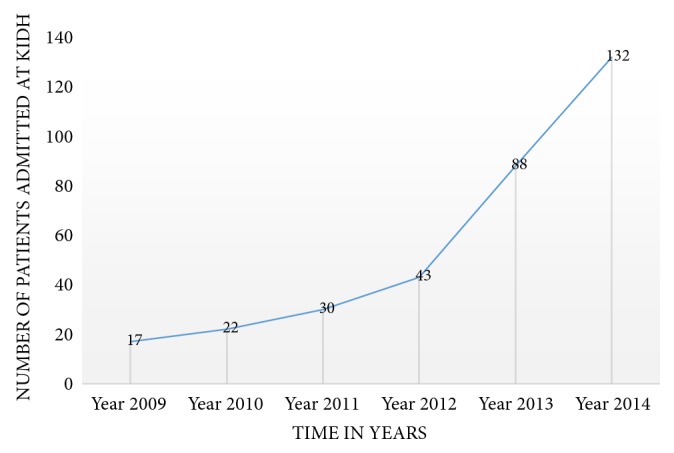
Trend showing increase in patients over years (2009-2014).

During the study period data for a total of 332 patients who were admitted at KIDH between 2009 and 2014 was collected. About two-thirds (221 (67%)) of the patients were males, and 317 (95.48%) were above 18 years of age. About half of the patients (161 (48.5%)) had been residing in Dar es Salaam. The mean and median ages of the study population were 36.9 years (SD 13.59) and 36 years (IQR of 27.5 to 46 years), respectively (Table 1). The number of patients being treated at KIDH has been steadily increasing from 2009 when the MDRTB program started (Figure 1).

**Figure 2:**
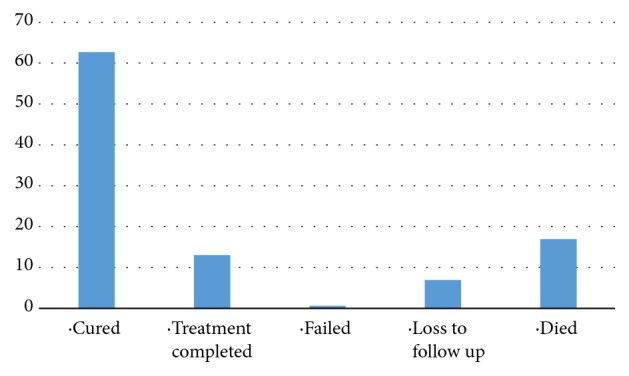
Percentages of patients' treatment outcomes (N=332).

**Figure 3:**
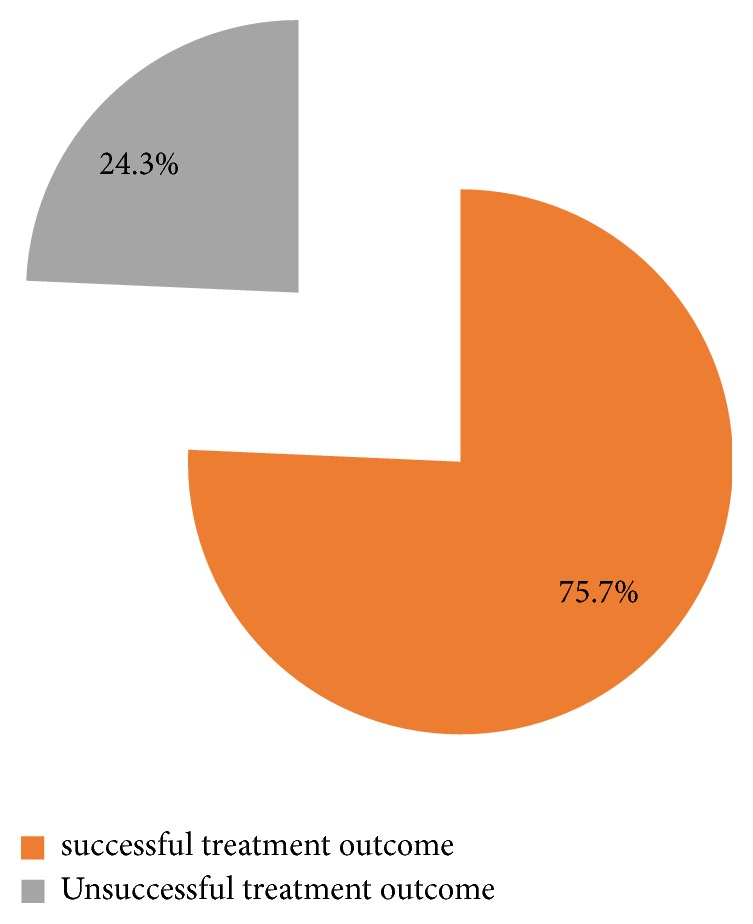
The proportion of successful and unsuccessful treatment outcomes in five years' time. (2009-2014).

**Figure 4:**
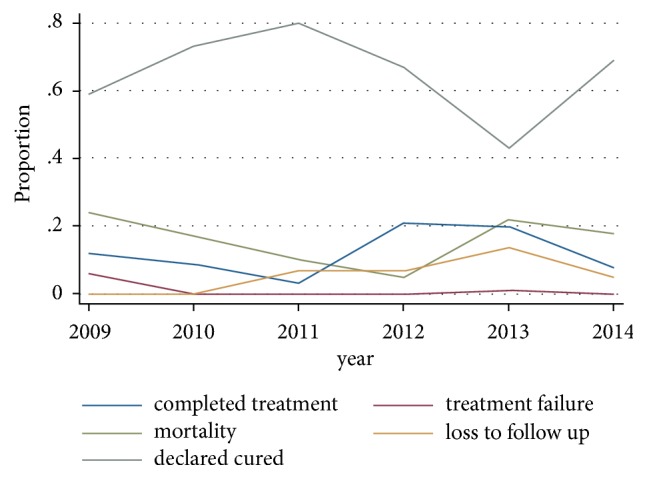
Trends of proportions of patients who were cured, completed treatment, were lost to follow-up, and died from 2009 to 2014.

The majority of patients (273 (82.2%)) had previous TB history while 95 patients (28.6%) and 68 patients (20.5%) had history of alcohol use and smoking, respectively (Table 2). More than one-third of the patients were HIV positive (116 (34.6%)). Out of 212 patients,109 patients (51.42%) had a BMI below 18kg/m^2^. A total of 279 X-ray films were available for interpretation and 64 (19.3%) of them had showed cavitary lesions (Table 2). Most of the patients had TB strains resistant to rifampicin (202 patients (60.8%)) and 130 patients (39.2%) were resistant to isoniazid (Table 3). Two hundred and eight patients (62.7%) were cured: 43 (13%) completed treatment, while only 2 (0.6%) had failed treatment (Figure 2) and a total of 252 patients (75.6%) had successful treatment outcome (Figure 3). More than 50% of all patients who have been treated at KIDH were declared cured each year (Figure 4). After multivariate analysis, it was found that low BMI (below 18 kg/m^2^) is significantly associated with reduced chance of being cured, aOR=0.59 (95% CI 0.39-0.88), p value of 0.044, while presence of cavitary lesions on chest X-rays had association with being cured of MDRTB (aOR=1.89 (95% CI 1.27-2.81), p value 0.002). Resistance to streptomycin was positively associated with cure, while resistance to ethambutol was negatively associated with cure (aOR =4.67 (95% CI 1.53-14.32), p value 0.007 and 0.34 (95% CI 0.12-0.96), and p value 0.041, respectively), (Table 4). No independent factors were found to be associated with MDRTB treatment completion (Table 5). Resistance to streptomycin and resistance to ethambutol had negatively (aOR =0.14 (95% CI 0.02-1.14), p value 0.066) and positively (aOR =4.65 (95% CI 0.88-24.56), p value 0.070) borderline association with loss to follow-up outcome, respectively (Table 6). Smoking habit and presence of cavitary lesions on chest radiographs were independent factors that became positively and negatively associated with mortality after multivariate analysis, with aORs of 2.31 (95% CI 1.35- 5.01), p value of 0.043) and 0.55 (95% CI 0.34-0.91), p value 0.019), respectively, (Table 7).

The sociodemographic characteristics of MDRTB patients admitted at KIDH between 2009 and 2014 (N=332)

## 4. Discussion

This study has found that the absolute numbers of MDRTB patients receiving treatment at KIDH since 2009 has been increasing as well as the number of patients being cured (a total of 208 patients were cured, 62.7% of all patients) (Table 2). Similar findings were observed in South Africa whereby the cure rate was 79% [21]. This could be due to increasing awareness of the MDRTB disease over the time and stability of the services provision by the hospital. Most of the patients, 317 (95.48%) were above 18 years of age, indicating that in Tanzania the most productive age category is the mostly affected with the disease. Forty-eight percent of all these patients were coming from Dar es Salaam, which is the busiest and overcrowded city in the country indicating the potential increased risk of disease transmission in overcrowded cities. Only 116 patients (34.9%) were HIV positive and 273 patients (82.2%) had positive previous TB treatment (Table 2). Only 2 patients (0.6%) had treatment failure during the entire time indicating both effective drug regimens and a stable health system (Figure 2). None of the patient had a missing treatment outcome, also a good indication of the regimen effectiveness and stable health system.

After multivariate analysis, presence of cavities in chest radiographs was associated with cure aOR 1.89. This is different from other findings, for example, a study by Kempker found that cavities were associated with increased drug resistance [22] as well as poor prognosis [11]. It could as well be due to the fact that cavities in the chest radiographs are more common among MDRTB patients compared to drug-sensitive TB patients [23]. But also variations in chest X-rays interpretation could also result in overdetection of cavities among MDRTB patients in our context, as found in Western Europe [24].

Low BMI was found to be significantly related to treatment cure in this study aOR 0.59. Other studies such as the one done by Kwak did not find a significant association between BMI and MDRTB treatment success [8], while a study done by Tang and colleagues found a significant association between low BMI (less than 18.5kg/m^2^) and poor treatment [11].

Resistance to streptomycin and ethambutol was each associated with MDRTB treatment cure. Our study found out that patients with TB strains resistant to streptomycin had an increased chance of being cured (aOR 4.67) while resistance to ethambutol was associated with decreased chance of being cured (aOR 0.34). Other studies have found other MDRTB drug, such as ofloxacin resistance which was inversely associated with MDRTB cure [18].

Dhingra identified the other factors as predictors of MDRTB treatment cure: weight gain at six months, sputum culture conversion, radiological improvement during treatment, resistance to strains less than or up to three antituberculosis drugs, use of less than or up to three second line drugs, and no change of treatment regimen during MDRTB treatment [25].

None of the independent factors predicted completion of treatment even after multivariate analysis but 43 patients (13%) had treatment completion as an outcome. The reason for not testing sputum culture and smear after completion of MDRTB treatment could be due to high number of consecutive negative sputum smear and culture at the final months during continuation phase of treatment, as well as the cost involved in these terminal follow-up visits. Most of these patients were discharged after an intensive phase of treatment and would be required to come back monthly for sputum cultures and smears as well as taking their monthly medications during the continuation phase of treatment. The last visit would only require them to come for sputum test to determine their cure status, without provision of any additional medications. Most of these patients would regard themselves as cured after several negative sputum smear and culture results towards the end of treatment and so would miss the last visit. Though none of the predictors had any association with completion of treatment, a study in South Africa found that HIV patients on ARVs were more likely to complete MDRTB treatment compared to HIV negative patients [12]. Both cure and treatment completion are defined by the WHO as successful treatment outcomes [3]. Other factors related to successful treatment outcomes include intensive treatment of adverse effects, nutritional supplementation, adherence interventions, and collaboration between the Ministry of Health and other nongovernmental organizations [26]. Others include the use of fluoroquinolones or bacteriostatic drugs [27], also having no previous history of TB treatment [28], BMI > 18.5kg/m^2^ [7], and individualized treatment [9].

None of the predictors had a significant association with loss to follow-up, but other studies have found other factors that were related to loss to follow-up such as alcohol abuse and developing adverse reactions while protective factors include receiving assistance from TB program, patients' better knowledge of TB, and trust in treating physicians and nurses [10]. A study by Javaid and colleagues found that living in rural areas had an increased risk of being loss to follow-up [18].

Smoking, which lowers immunity against TB, was associated with mortality (aOR 2.31). Similar findings have been observed by Mollel and colleague whereby cigarette smoking and HIV positive status were both positively associated with MDRTB mortality [29]. Presence of cavities on chest X-rays was inversely associated with mortality. This is different from other studies such as that done by Kempker [22] and Tang [11]. Other predictors of MDRTB mortality include drug resistance pattern [30], diabetes history [31], anemia, positive sputum smear, hepatitis and drug use, resistance to ofloxacin [18], HIV positive infection, and low CD4 count [29]. A study in Egypt indicated that being diabetic is associated with unsuccessful MDRTB treatment outcome [32]; this could be related to the effect of diabetes on delaying sputum culture conversion in TB patients [33], leading to treatment failure and death [34].

Being a retrospective study, this study had some limitations, including missing data for some parameters such as smoking and alcohol use, but also diabetic patients were few. This may have had an impact on the results belonging to this category.

## 5. Conclusion

While streptomycin resistance and presence of cavity on chest radiographs are positively associated with MDRTB cure, ethambutol resistance and low BMI were inversely associated with MDRTB cure. Smoking and presence of cavities in chest radiographs were positively and negatively associated with MDRTB mortality, respectively. The study recommends, improving nutritional status of MDRTB patients, widespread antismoking campaign, and close follow-up of patients with ethambutol resistance.

## Figures and Tables

**Table 1:** Patients' sociodemographic characteristics (N=332).

Patient's characteristics	N=332 (% of total)
Age (mean =36.9 years (SD 13.59), median =36 years)	
(i) Below 18	15(4.52)
(ii) Above or equal 18	317(95.48)

Sex	
(i) Male	221(66.6)
(ii) Female	111(33.4)

Residence	
(i) Dar es Salaam	161(48.5)
(ii) Other regions	171(51.5)

Marital status	
(i) Divorced	14(4.20)
(ii) Married	126(38.0)
(iii) Single	176(53.0)
(iv) Widow/widower	16(4.82)

**Table 2:** Patients' clinical and radiological features (N=332).

Characteristics	N(% of total)
BMI (N=312)	
(i) Below 18.5 kg/m^2^	109(51.42)
(ii) Above or equal to 18.5 kg/m^2^	103(48.58)
HIV status	
(i) Positive	116(34.9)
(ii) Negative	216(65.1)
History of previous TB treatment (N=329)	
(i) Yes	273(82.2)
(ii) No	56(16.9)
Smoking history (N=177)	
(i) Yes	68(20.5)
(ii) No	109(32.9)
Alcohol use (N=189)	
(i) Yes	95(28.6)
(ii) No	94(28.3)
Cavitary lesions on Chest x-ray radiography (N=279)	
(i) Yes	64(19.3)
(ii) No	215(64.8)
Diabetes (N=319)	
(i) Yes	5(1.5)
(ii) No	314(94.6)

**Table 3:** Patients' drug susceptibility test (DST) results.

Drug name	N(%)
Isoniazid(N=141)	
(i) Resistant	130(39.2)
(ii) Susceptible	11(3.3)

Rifampicin (N=207)	
(i) Resistant	202(60.8)
(ii) Susceptible	5(1.5)

Streptomycin(N=114)	
(i) Resistant	80(24.1)
(ii) susceptible	34(10.2)

Ethambutol (N=110)	
(i) Resistant	73(22.0)
(ii) Susceptible	37(11.1)

**Table 4:** Adjusted OR after multivariate analysis for predictors of treatment outcome of cure.

Characteristics	Cured	Other treatment categories	aOR (95% CI)	p-value
	N (%)	N (%)		
Cavitary lesions on CXR (N=279)				
Yes	45(70.3)	19(29.7)	1.89(1.27-2.81)	0.002
No	143(66.5)	72(33.5)		

BMI				
Below 18.5	9(60.0)	6(40.0)	0.59(0.39-0.88)	0.044
Above or equal 18.5	199(62.8)	118(37.2)		

Residence				
Dar	105(65.2)	56(34.8)	1.03(0.58-1.81)	0.922
Other	103(60.2)	68(39.8)		

Positive baseline sputum culture (N=253)				
Yes	105(68.2)	49(31.8)	1.55(0.79-3.01)	0.200
No	62(62.6)	37(37.4)		

HIV status (N=208)				
Yes	71(61.2)	45(38.8)	1.03(0.57-1.86)	0.933
No	137(63.4)	79(36.6)		

Positive baseline sputum smear (N=253)				
Yes	105 (68.2)	49 (31.8)	0.62(0.30-1.29)	0.203
No	62 (62.6)	37 (37.4)		

Isoniazid (N=141)				
Resistant	87(66.9)	43(33.1)	1.03(0.47-2.26)	0.948
susceptible	7(63.6)	4(36.4)		

Rifampicin (N=207)				
Resistant	128(63.4)	74(36.6)	0.77(0.37-1.59)	0.479
Susceptible	4(80.0)	1(20)		

Streptomycin (N=114)				
Resistant	51(63.8)	29(36.3)	4.67(1.53-14.32)	0.007
susceptible	28(82.4)	6(17.6)		

Ethambutol (N=110)				
Resistant	50(68.5)	23(31.5)	0.34(0.12-0.96)	0.041
susceptible	25(67.6)	12(32.4)		

**Table 5:** Adjusted OR after multivariate analysis for predictors of treatment outcome of completed treatment.

Characteristics	Completed treatment	Other treatment categories	aOR(95% CI)	p-value
	N (%)	N (%)		
Baseline sputum smear				
(i) Yes	32(15.2)	178(84.8)	2.09(0.66-6.61)	0.210
(ii) No	7(8.0)	81(92.0)		

Baseline sputum culture				
(i) Yes	22(14.3)	132(85.7)	1.25(0.47-3.29)	0.654
(ii) No	9(9.1)	90(90.9)		

HIV status				
(i) Yes	15(12.9)	101(87.1)	0.99(0.43-2.29)	0.977
(ii) No	28(13.0)	188(87.0)		

Isoniazid				
(i) Resistant	16(12.3)	114(87.7)	1.17(0.41-3.33)	0.765
(ii) susceptible	2(18.2)	9(81.2)		

Rifampicin				
(i) Resistant	26(12.9)	176(87.1)	1.56(0.55-4.43)	0.405
(ii) Susceptible	1(20.0)	4(80.0)		

Streptomycin				
(i) Resistant	9(11.3)	71(88.8)	0.31(0.08-1.26)	0.101
(ii) Susceptible	4(11.8)	30(88.2)		

Ethambutol				
(i) Resistant	7(9.6)	66(90.4)	2.49(0.68-9.03)	0.166
(ii) susceptible	6(16.2)	31(83.8)		

**Table 6:** Adjusted OR after multivariate analysis for predictors of treatment outcome of loss to follow-up.

Characteristics	Loss to follow up	Other treatment categories	aOR(95% CI)	p-value
	N (%)	N (%)		
Residence				
(i) Other	7(4.3)	154(95.7)	2.41(0.84-6.91)	0.102
(ii) Dar	16(9.4)	155(90.6)		

Diabetes				
(i) Yes	1(20.0)	4(80.0)	0.29(0.04-2.08)	0.216
(ii) No	19(8.9)	295(91.1)		

Smoking				
(i) Yes	6(7.7)	72(92.3)	0.74(0.32-1.77)	0.509
(ii) No	5(4.6)	104(95.4)		

HIV status				
(i) Yes	8(6.9)	108(93.1)	0.96(0.34-2.76)	0.943
(ii) No	15(6.9)	201(93.1)		

Alcohol use				
(i) Yes	9(9.5)	86(90.5)	1.61(0.72-3.56)	0.244
(ii) No	5(5.3)	89(94.7)		

Isoniazid				
(i) Resistant	9(7.4)	121(92.6)	1.22(0.32-4.68)	0.768
(ii) Susceptible	1(9.1)	10(90.9)		

Rifampicin				
(i) Resistant	14(6.9)	188(93.1)	0.93(0.25-3.44)	0.909
(ii) Susceptible	0(0.0)	5(100.0)		

Streptomycin				
(i) Resistant	7(8.8)	73(91.2)	0.14(0.02-1.14)	0.066
(ii) Susceptible	1(2.9)	33(97.1)		

Ethambutol				
(i) Resistant	4(5.5)	69(94.5)	4.65(0.88-24.56)	0.070
(ii) Susceptible	4(10.8)	33(89.2)		

**Table 7:** Adjusted OR after multivariate analysis for predictors of treatment outcome of mortality/death (N=332).

Characteristics	Died	Other treatment categories	aOR(95% CI)	p-value
	N (%)	N (%)		
Cavitary lesions on CXR (N=279)				
(i) Yes	8(12.5)	56(87.5)	0.55(0.34-0.91)	0.019
(ii) No	29(13.7)	182(86.3)		
Smoking (N=177)				
(i) Yes	24(31.6)	52(68.4)	2.31(1.35-5.01)	0.043
(ii) No	14(12.8)	95(87.2)		
Baseline positive sputum smear (N=268)				
(i) Yes	33(16.0)	173(84.0)	1.24(0.48-3.21)	0.661
(ii) No	13(14.9)	74(85.1)		
Baseline positive sputum culture (N=253)				
(i) Yes	16(10.5)	136(89.5)	0.55(0.23-1.34)	0.190
(ii) No	17(17.3)	81(82.7)		
HIV status				
(i) Yes	22(19.5)	91(80.5)	1.10(0.49-2.50)	0.815
(ii) No	34(15.9)	180(84.1)		
Diabetes (N=319)				
(i) Yes	1(20.0)	4(80.0)	1.49(0.33-6.71)	0.609
(ii) No	52(16.8)	258(83.2)		
Isoniazid				
(i) Resistant	17(13.3)	111(86.7)	0.77(0.24-2.46)	0.661
(ii) susceptible	1(9.1)	10(90.9)		
Rifampicin				
(i) Resistant	33(16.7)	165(83.3)	1.27(0.50-3.22)	0.614
(ii) Susceptible	0(0.0)	5(100.0)		
Streptomycin				
(i) Resistant	12(14.8)	67(85.2)	0.54(0.10-2.78)	0.459
(ii) Susceptible	1(3.0)	32(97.0)		
Ethambutol				
(i) Resistant	11(15.3)	61(84.7)	0.97(0.20-4.68)	0.974
(ii) Susceptible	2(5.6)	34(94.4)		

## Data Availability

The data used to support the findings of this study are available from the corresponding author upon request and permission from Kibong'oto Infectious Diseases Hospital (KIDH).
